# Supplementary figures and images for: Genetic diversity and neutral selection in Plasmodium vivax erythrocyte binding protein correlates with patient antigenicity
Source: PLoS Negl Trop Dis. 2020 Jul 9;14(7):e0008202. doi: 10.1371/journal.pntd.0008202 (PMC7347095; doi:10.1371/journal.pntd.0008202)

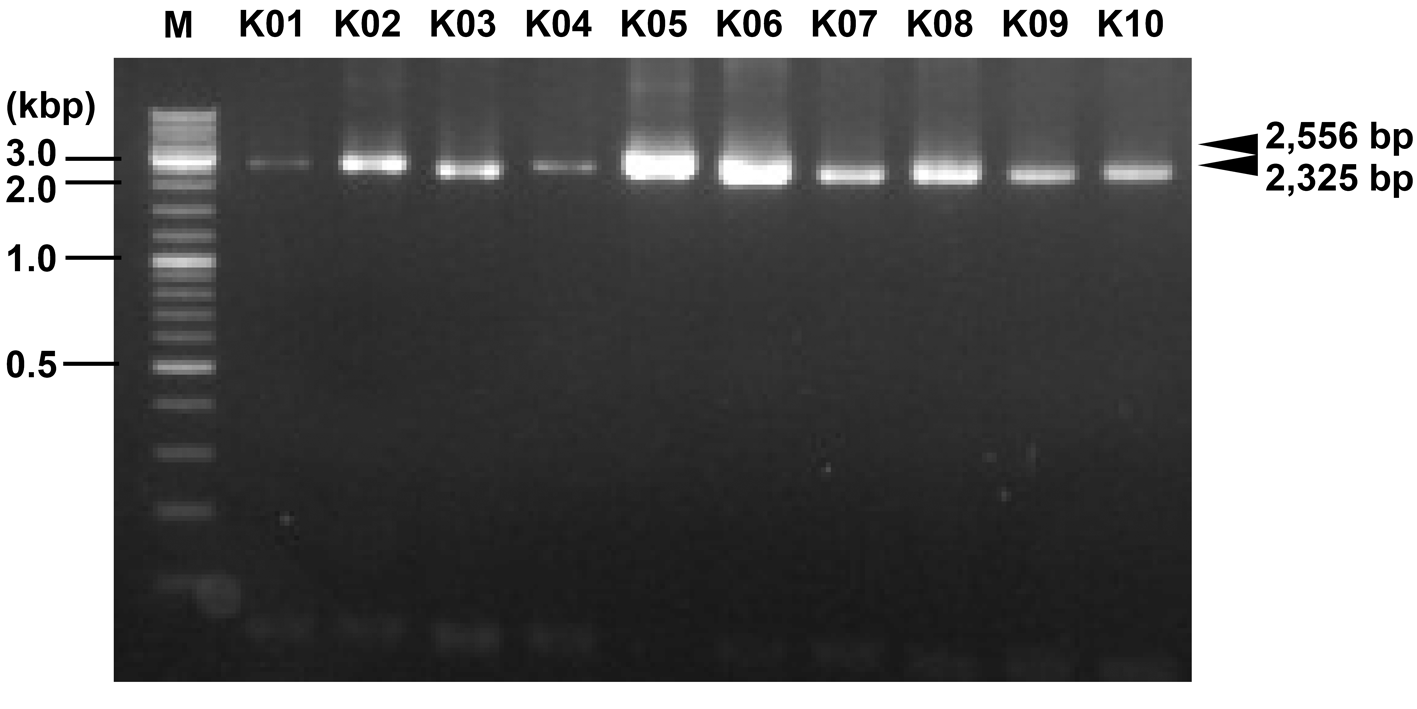

Supplement: S1 Fig — PvEBP-ecto gene in ROK samples shown different amplicon size for approximately 2,556 bp for K_01, 02, 04, and 05 and 2,325 bp for K_03, 06, 07, 08, 09, and 10. (TIF) [file pntd.0008202.s001.tif]
