# Supplementary material for: Genetic diversity and neutral selection in Plasmodium vivax erythrocyte binding protein correlates with patient antigenicity
Source: PLoS Negl Trop Dis. 2020 Jul 9;14(7):e0008202. doi: 10.1371/journal.pntd.0008202 (PMC7347095; doi:10.1371/journal.pntd.0008202)
Supplement: Attachment — Submitted filename: (R1-PNTD) Response_Han_2019.docx [file pntd.0008202.s003.docx]

**Reviewer comments: 
Reviewer #1 (Remarks to the Author):** 

**Methods**
Reviewer #1: I suggest to comment more about the neutrality tests, sometimes significant negative values simply indicate population expansion rather than purifying selection (see Molecular biology and evolution 30: 2050-2064 and similar types of work). I also suggest some form of MK tests to be applied, together with estimating Ds Dn. I believe that a more detailed comparison of different neutrality tests will benefit this work.

Response: Thank you for your comments. We performed more neutrality tests of MK tests and Dn/Ds ratio comparison. The method added on page 7: lane 151-158.

--------------------

**Results**
Reviewer #1: This is an interesting report. Usually immunity is related to balancing selection and the authors did not find evidence of it using these neutrality tests. Please provide the sequences accession numbers.

Response: Thank you for your comments. This gene have an important role for parasite invasion to the proper host cell however, the exact immune profile and function need to be confirm at the future. The sequences are uploaded at NCBI (accession number: MN853168 - MN853300) and added in manuscript page 6: lane 137-138.


--------------------

**Conclusions**
Reviewer #1: Need to discuss more the limitations of neutrality tests in this context.

Response: Thank you for your comments. We added neutrality tests using MK test and D_n_/D_s_ test result in the result section (page 9-10: lane 227-234, and table 2). Additionally, page 12: lane 303-305 and page 13: lane 315-316 added discussion of neutrality.

--------------------

**Editorial and Data Presentation Modifications?**
Reviewer #1: Minor revision (need to deposit data in the GenBank or other major database).

--------------------

**Summary and General Comments**
Reviewer #1: This is an interesting report. Usually immunity is related to balancing selection and the authors did not find evidence of it using these neutrality tests. However, I suggest to comment more about it, sometimes significant negative values simply indicate population expansion rather than purifying selection (see Molecular biology and evolution 30: 2050-2064 and similar types of work). I also suggest some form of MK tests to be applied, together with estimating Ds Dn. I believe that a more detailed comparison of different neutrality tests will benefit this work. The accession numbers of the sequences should be provided.

Response: We really appreciated for reviewer comments to improve evidence of *pvebp* gene neutrality evidence. All comments were valuable to improve this manuscript quality. We performed additional test and updated to manuscript following reviewer comments.
